# A case study on optimizing industrial air conditioning with thermal solar energy in Egypt

**DOI:** 10.1016/j.heliyon.2024.e34774

**Published:** 2024-07-18

**Authors:** Mohammed A. Ebaid, Tamer A. Mohamed, Hesham Safwat

**Affiliations:** Department of Mechanical Engineering, The British University in Egypt, 11837, El Sherouk City, Egypt

**Keywords:** Solar, Adsorption, Industrial, Energy, Savings, Optimization

## Abstract

This study investigates the feasibility of implementing a solar-assisted adsorption chiller in an industrial building at the Oriental Weavers International factory located in 10th of Ramadan City, Cairo, Egypt. The objective is to replace an inefficient split air conditioning system currently used to cool the Jacquard units during carpet manufacturing. The research begins by analyzing the performance of the existing cooling system to establish a baseline. It then explores the potential energy savings achievable by replacing the current system with a solar-assisted adsorption chiller. The existing oversized boiler will serve as an auxiliary heater for the new system. TRNSYS simulation tools are employed to model the building, simulate its thermal performance, and develop a solar-assisted cooling system. A parametric analysis investigates the impact of varying collector area and hot/cold-water storage tank volumes on key energy performance indicators. This analysis aims to determine the optimal component sizes needed for efficient system operation. Results indicate that a collector area of 90 m^2^ offers the optimal balance between performance and cost. There are minimal benefits to increasing the collector area beyond 100 m^2^. Larger hot water storage tanks demonstrate reduced outlet temperatures, reaching a maximum solar fraction at a capacity of 4 m³. The impact of cold-water storage tank volume on the system is minimal. The economic assessment reveals a payback period of 7.6 years, an Internal Rate of Return (IRR) of 14.3 %, and a Return on Investment (ROI) of 34.5 % over a 10-year period, indicating the financial viability of the proposed system. Furthermore, the solar-assisted adsorption chiller system has the potential for substantial environmental benefits. The system has the capacity to reduce CO2 emissions by up to 7200 metric tons. This highlights not only the technical feasibility of the system but also its economic and environmental advantages.

## 1 Introduction

Egypt, with its hot and arid climate, is confronted with the daunting task of meeting the rising energy demand, primarily driven by the increasing cooling needs in buildings. From 1990 to 2020, the residential sector emerges as the predominant power consumer, constituting 42 % of total electricity consumption in Egypt as of 2020. Following closely, the industrial sector contributes 28 % to Egypt's overall electricity usage. In the past decade, the industrial sector has experienced a notable 5 % increase in electricity consumption, rising from 146,527 TJ in 2008 to 153,417 TJ in 2020 [1]. Air conditioning alone contributed to 5 % of the total electricity consumption, emphasizing the need to address cooling energy demands [2]. Egypt's abundant solar radiation suggests solar thermal energy as a sustainable alternative. The global building sector, responsible for 35 % of total energy consumption and 38 % of CO_2_ emissions in 2020, plays a substantial role [3]. In 2022, space cooling demand witnessed the largest increase across all building types, raising concerns about peak electricity demand and the potential for a 40 % increase in global electricity demand for space cooling by 2030 [3]. In response to escalating energy demand and climate concerns, renewable energy technologies, particularly solar energy for cooling, offer a viable solution. Solar technologies provide an economically and ecologically viable alternative to fossil fuels, contributing to global efforts to mitigate fossil fuel shortages. Despite initial challenges, technological advancements have led to cost reductions and increased efficiency in solar systems, resulting in a significant decline in the levelized cost of solar energy. The International Energy Agency projects that solar cooling will constitute 17 % of global air conditioning by 2050 [4], although challenges remain, including high initial investment costs for thermal solar cooling systems using absorption machines, leading to extended payback times that may surpass their operational lifespan. Historically, absorption and adsorption chillers have relied on natural gas or heat derived from industrial waste for power. Recent studies have shown the potential of harnessing solar thermal energy as a viable alternative to power absorption/adsorption chillers.

Numerous works in literature delve into the modelling and simulation of solar-assisted absorption and adsorption systems, categorizing them based on their applications in residential, commercial, and industrial settings. For residential applications, many studies were conducted. For instance, Salameh et al. [5] devised a solar-powered absorption cooling system for a UAE residence using LiBr–H_2_O, with TRNSYS software employed for simulation and optimization. The system achieved a Coefficient Performance (COP) of 0.793. Optimization results indicated that the UAE's latitude favors a specific tilt angle for the evacuated tube. The optimal collector area was 40 m^2^, and the optimal hot water storage tank volume was 1 m^3^, resulting in 0.73 solar percentages. Life cycle analysis revealed that the solar-powered system incurred a 43.2 % higher cost, consumed 8.5 % more energy, and had an 8.7 % carbon footprint compared to conventional vapor compression systems. Also, Motamedi et al. [6] conducted a research study evaluating the economic feasibility of a solar-powered adsorption cooling system. The system was theoretically presented with the solar percentage calculated initially. Rahman et al. [7] studied the design, modelling, and simulation of an absorption solar air-conditioning system using TRNSYS. The system showed promise in meeting domestic air-conditioning demand in Lahore, Pakistan, with an average collector outlet temperature of 78 °C, 71,065 kJ/h useable energy generation, and average room temperatures of 27 °C, 30 °C, and 19 °C in three rooms. A solar-powered absorption cooling system was simulated using TRNSYS in various Turkish cities by Altun and Kilic [8]. Izmir was deemed the most suitable, with a payback period of 10.7 years, while Trabzon exhibited the longest payback period and highest levelized cost of cooling among the cities. Furthermore, Ali [9] compared residential-scale solar thermal, a silica gel-water adsorption chiller, and an air-conditioning system to a conventional system in hot, dry climates for performance, energy efficiency, cost competitiveness, and global warming. The study, conducted in Assiut, Egypt, demonstrated the viability of an 8-kW off-grid PV-driven DC air conditioning system. The solar thermal cooling systems exhibited lower energy consumption and cost per kW of cooling compared to vapor AC-driven air-conditioning systems. The off-grid PV-driven DC air conditioning system presented as a suitable option for residential use in hot, dry climates due to its low cost per kW of cooling, 0 % grid energy demand, and low Total Equivalent Warming Effect (TEWI) value. Yang et al. [10] investigated solar assisted air source heat pumps in UK climates. This study models and simulates three types of these heat pumps (serial, parallel, and dual-source indirect expansion) using TRNSYS software in London. Each configuration is designed to provide space heating and 300 L of hot water daily for a typical single-family home. Results indicate that the serial configuration achieves the highest seasonal performance factor of 5.5 but requires larger solar collector and storage tank sizes. In contrast, the dual-source and parallel configurations achieve slightly lower seasonal performance factors of 4.4 and 4.5, respectively, with smaller equipment requirements. The study emphasizes the significant role of the air source component in providing heat and ensuring system stability. With seasonal performance factors exceeding 4.4, these systems show potential for application in regions with moderate solar radiation. Economic evaluations suggest that the parallel and dual-source configurations offer feasible alternatives to conventional gas-boiler heating systems. In another review article, Yang et al. [11] examines research detailing five typical and five advanced Solar Assisted Air Source Heat Pump (SAASHPs), along with discussions on three types of solar thermal collectors, three thermal energy storage methods, and ten defrosting techniques. Studies indicate that SAASHPs show promise for providing both space heating (SH) and hot water (HW) in residential settings. Researchers primarily focus on mid-latitude regions (20°–50°) where SH is necessary in winter and HW is required year-round, under moderate solar irradiance and temperate climates (−15 °C–30 °C). Future research directions include advancing multifunctional SAASHP designs and exploring their applicability in high-latitude areas, especially through advanced Indirect Expansion (IX) SAASHPs with improved solar collectors and thermal storage technologies. Most SAASHPs achieve Coefficient of Performance (COP) values between 2 and 6. Notably, dual-source IX-SAASHPs typically achieve COP values below 3.5, while hybrid, serial IX, advanced Direct Expansion (DX), and dual-source DX-SAASHPs can achieve COP values up to 6. DX-SAASHPs and advanced IX-SAASHPs exhibit significant potential, achieving COP values exceeding 6 and reaching up to 10.

For commercial applications of solar absorption, in their investigation, Noferesti et al. [12] utilized the TRNSYS simulation tool to model a solar absorption air conditioning system tailored for an Iranian office building. The model considered critical factors such as the system's thermal storage capacity, the efficiency of the solar collectors employed, and the thermal load characteristics of the building. The outcomes indicated that the system exhibited a cooling capacity of 10.2 kW and a performance coefficient of 0.52. Notably, the solar-assisted absorption chiller, when equipped with Evacuated Tube, Glazed Painted, and Unglazed solar collectors, contributed to indoor temperature improvements of up to 3.08 °C, 1.68 °C, and 1.46 °C, respectively, during regular office working hours. Furthermore, the study demonstrated substantial reductions in power consumption, reaching up to 84 %, 64 %, and 48 %, respectively, when compared to the building's existing cooling system. This underscores the considerable potential of employing solar-assisted absorption chillers to enhance energy efficiency and create a more comfortable indoor environment. In 2020, Martinez et al. [13] developed a simplified TRNSYS simulation model for a 35 kW Solar Cooling Demonstration Facility intended for a hotel in Spain. The study explored various configurations, including different collector field surfaces, hot water storage tank capacities, and absorption machine driving temperatures. The optimal system configuration, maximizing energy transfer to the absorption chiller, was identified by aligning with the lowest values of driving temperature (75 °C) and specific storage volume (15 L/m^2^). Despite achieving an annual savings of 1515 euros compared to an electric compression chiller, the economic viability was constrained by the substantial initial investment of 3000 euros per kW of cooling capacity required for the thermal sun cooling facility. This underscores the economic challenges associated with implementing solar cooling solutions in certain contexts. Utham et al. [14] conducted a modeling and simulation study using TRNSYS to design a solar absorption cooling system for the GERMI office building in Gandhinagar, Gujarat, India, covering an area of 1000 square meters. The system utilized evacuated tube collectors to power a single-effect absorption chiller, aiming to fulfill the building's cooling requirements. The study also delved into the impact of storage tank volume, collector area, and collector slope through parametric optimization. The results of the optimization revealed that, for the simulated solar-based absorption system, an arrangement of 35 m^2^ of evacuated tube collectors with an inclination of 24.2° and a storage tank volume of 0.5 m^3^ was necessary to meet the cooling demands efficiently.

However, when it comes to industrial application studies, research is lacking, with only a few studies being found. One of which was conducted by Mehmood et al. [15], a comprehensive comparison is made between the energy, economic, and environmental performance of solar-powered absorption cooling systems and electrically driven water-cooled vapor compression systems. The study delves into investigating the parameters influencing the efficiency of solar-driven vapor absorption systems. Using TRNSYS, a simulation of both systems' operations is carried out to meet the cooling needs of an industrial manufacturing plant in Lahore, Pakistan. The evaluation of each system considers primary energy savings, initial investment, operational costs, and carbon footprint indicators. Additionally, a Python-based parametric code is developed and integrated with TRNSYS to analyze the impact of various parameters on the performance of solar-driven vapor absorption chillers. These parameters encompass the solar field size, storage tank volume, optimal annual and monthly collector angles, as well as the flow rate in the solar field. The results suggest that adjusting the collector angle monthly in accordance with the sun's position can increase energy collection by approximately five percent compared to a fixed-angle approach. Notably, electrically driven vapor compression cooling systems exhibit higher operational costs and environmental risks despite their lower upfront expenses. Conversely, solar thermal systems demonstrate reduced operational costs and emissions, but their economic sustainability may necessitate significant capital cost reductions or government subsidies. This study contributes valuable insights for optimizing solar-driven cooling systems and understanding their economic and environmental implications. Allouhi et al. [16] conducted a study which introduces an optimization procedure and simulation of a centralized solar heating system designed to supply hot water to four processes with varying temperature requirements and load profiles. The case study focuses on evaluating a Moroccan milk processing company based in Casablanca, employing the life cycle cost method to determine the optimal sizing of key design parameters for decision-making. The findings suggest that installing 400 m^2^ of evacuated tube collectors tilted at a 30° angle, connected to a 2000-L storage tank, could result in maximum life cycle cost savings of 179 thousand USD per year, meeting a total annual heat demand of 528.23 MWh. In this optimized configuration, the system achieves an annual solar fraction of 41 % and a payback period of 12.27 years. Additionally, the system has the potential to annually reduce approximately 77.23 tons of CO_2_ equivalent greenhouse gas emissions.

Most existing literature primarily explores the feasibility of solar absorption air conditioning solutions for residential and commercial purposes. However, there is a noticeable gap in research regarding industrial applications in Egypt. Consequently, this study was undertaken with the principal objective of examining the feasibility of substituting conventional electrical split air conditioning units with a solar-assisted adsorption chiller in an industrial building subjected to Egyptian climatic conditions. The study also aims to evaluate the energy performance, power consumption, and potential energy savings associated with this transition.

## 2 Research methodology

In this current work, the feasibility of integrating a solar-assisted adsorption chiller into an industrial building in Cairo, Egypt is examined. The objective was to replace the existing split-unit cooling system with a solar adsorption chiller during periods when solar energy is sufficient to meet the system's energy requirements. This shift in power consumption not only conserves energy resources, such as fossil fuels, but also mitigates the emission of harmful environmental pollutants.

### 2.1 Existing cooling system

The initial assessment will focus on the industrial building specifications and cooling demand requirements, as well as the power consumption of the existing cooling system, which consists of 32 split air conditioning units, with each Jacquard Room has two air conditioning units for cooling the Jacquard unit at set point of 24 °C. Technical specifications for the existing cooling system can be seen in Table 1.

**Table 1:** Power P042CMXV split unit technical specifications.

Brand	POWER
Model	P042CMXV
Power Supply	220 V/1 Ph/50Hz
Capacity (BTU/h)	42000
Power Input (W)	4200
EER	10
Manufacturing date	2001

Annual power consumption of the existing system is calculated using equation (1) below, considering degradation in EER:

(1) $${EER}_{degradation}={EER}_{nominal}*{\left(1-M\right)}^{N}$$

where M is the maintenance factor (0.01 expertly maintained equipment; 0.03 unmaintained) and N is the equipment lifetime (years).

This process commenced with the creation of a building model in TRNbuild, enabling the simulation of the building's performance. The building model was subsequently incorporated into the TRNSYS simulation studio to ascertain the cooling requirements of the building. The peak cooling load was then utilized to ascertain the necessary capacity of the adsorption chiller. The proposed solar adsorption cooling system will then by modelled using TRNSYS simulation software to assess its necessary cooling capacity. The subsequent step involved modelling the remaining components, aligning with the technical specifications provided by manufacturers of commercially available products in the market to power the adsorption chiller. The final model of the proposed solar adsorption system will then by simulated for 8760 h (1 year) and the results will be parametrically analysed to determine the optimal system configurations.

### 2.2 Parametric analysis

Various critical design aspects, including collector field area, hot and cold-water storage tank volumes will go thorough parametric studies to evaluate their impact on the solar cooling system's overall performance by evaluating key energy performance indicators such as solar fraction. The primary aim of this analysis is to design a solar adsorption system tailored to the climatic conditions of Egypt, maximizing the practical energy output for the industrial building. Mathematical modeling serves to explore the physical system, identifying opportunities for performance enhancement. Simulation modeling proves valuable for assessing system behavior under varied design scenarios. This section employs parametric analyses to determine optimal sizes for key components of a solar adsorption cooling system, tailored to meet the cooling needs of an industrial building in Egypt. Table 2 illustrates the design parameters studied and their respective values employed in this section.

**Table 2:** Design parameters for parametric analysis.

Design Parameters	Unit	Value
Collector Field Area	m^2^	50–200
Hot Water Storage Tank Volume	m^3^	1–10
Cold Water Storage Tank Volume	m^3^	1–10

Collector field areas and water storage tank volume ranges shown in Table 2 were chosen according to the maximum free space available at site condition of the industrial building to accommodate these components.

### 2.3 Energy performance indicators

Assessing the energetic performance of a solar-adsorption chiller involves considering specific performance indicators, such as hot water storage tank outlet temperature, and solar fraction, which is the ratio of the thermal energy generated by the collector to the total energy (solar + auxiliary heater) as stated by Equation (2):

(2) $$S.F=\frac{\sum Q_{solar}}{\sum Q_{solar}+\sum Q_{aux}}$$

where SF is the solar fraction, Q_solar_ is the accumulated solar energy (kWh) and Q_auxiliary_ is the accumulated heat from auxiliary heating source (kWh).

### 2.4 Economic assessment

The economic feasibility of implementing a solar adsorption cooling systems requires a comprehensive economic assessment, which is heavily influenced by the substantial initial capital investments required for acquiring and installing the adsorption chiller and solar collectors. This economic evaluation aims to analyze the advantages and costs associated with project operations, with the goal of assessing the suitability and efficiency of resource utilization. To conduct a thorough economic evaluation, various methodologies are employed, each providing unique perspectives and insights. This research will utilize three primary methodologies to assess the economic feasibility of the proposed system: the payback period (PBP) approach, the net present value (NPV) and Internal Rate of Return (IRR) approach, and the return on investment (ROI) approach. Economic analysis considers critical assumptions and variables, as outlined in the results section of this study.

### 2.5 Payback period (PBP)

(3) $$\text{PBP}=\frac{C_{o}}{C_{F}}$$

where C_o_ is the total initial investment costs (USD $) and C_F_ is the annual cashflow (USD $).

### 2.6 Net present value (NPV)

(4) $$\text{NPV}=-C_{o}+\sum_{i=1}^{n}\frac{C_{i}}{{\left(1+r\right)}^{i}}$$

where C_i_ is the projected annual cashflow at year i (USD $), r is discount rate (%) and n is the project lifetime (years).

### 2.7 Internal rate of return (IRR)

(5) $$0=-C_{o}+\sum_{i=1}^{n}\frac{C_{i}}{{\left(1+\text{IRR}\right)}^{i}}$$

where IRR is the internal rate of return (%).

### 2.8 Return on investment (ROI)

(6) $$\text{ROI}=\frac{C_{\text{net}}}{C_{o}}*100\%$$

ROI: Return on Investment (%)

C_o_: Total Initial Investment Costs (USD $).

C_net_: Net return on investment (USD $).where C_net_ is net return on investment (USD $).

### 2.9 Environmental impact

An environmental assessment of solar cooling systems is undertaken, recognizing the growing significance of environmental issues in system design. To calculate CO_2_ emission reductions from implementing a solar-assisted adsorption chiller/cooling system, the baseline CO_2_ emissions generated by the existing cooling system needs to be determined. This involves assessing the energy consumption of the current system, which can be measured in kilowatt-hours (kWh) or British thermal units (BTUs). The energy savings achieved by the solar-assisted adsorption chiller are then compared to the baseline system through TRNSYS simulation software. The energy savings are then converted into CO_2_ emission reductions using a conversion factor. This factor represents the amount of CO_2_ emissions avoided per unit of energy saved. It accounts for the carbon intensity of the energy sources used to generate electricity. By multiplying the energy savings (in kWh or BTUs) by the conversion factor to calculate the amount of CO_2_ emissions avoided annually or over a specified period. The environmental impact of these CO_2_ emission reductions can then be assessed by quantifying the equivalent number of trees that would need to be planted to offset the emissions or comparing the reductions to regulatory standards or industry benchmarks for carbon footprint reduction. By following these steps, a robust and quantifiable assessment of the CO_2_ emission reductions achieved through the implementation of a solar-assisted adsorption chiller/cooling system. The calculation of the annual carbon dioxide emissions from a solar cooling adsorption chiller is calculated through the following equations:

(7) $${\text{CE}}_{E}=C_{E}*F_{E}$$

(8) $${\text{CE}}_{\text{NG}}=C_{\text{NG}}*F_{\text{NG}}$$

where CE_E_ is CO_2_ emissions from electricity (tCO_2_), CE_NG_ is CO_2_ emissions from natural gas (tCO_2_) C_E_ is the electricity consumption (kWh), C_NG_ is the natural gas consumption (mmBTU), F_E_ is the electricity grid emission factor (tCO_2_/MWh) and F_NG_ is the natural gas emission factor (tCO_2_/mmBTU).

## 3 TRNSYS software modelling

The simulation of a solar-assisted adsorption cooling system was executed using TRNSYS 18 simulation software. The Simulation Studio within TRNSYS is utilized for configuring a project graphically by connecting its components. The simulation engine incorporates a mathematical model for each component type, complemented by Proformas in the simulation studio as outlined in Table 3. Proformas provide a black-box description of a component's inputs, outputs, parameters, and more. The connections established between components enable the flow of information, allowing outputs from one component to serve as inputs for another [17].

**Table 3:** TRNSYS components, types, and mathematical model [17].

Component	Type	Mathematical model
Evacuated Tube Collector	Type 71	$\eta =a_{o}+a_{1}\frac{Tin-Tamb}{I_{T}}-\;a_{2}\frac{{\left(Tin-Tamb\right)}^{2}}{I_{T}}$
Auxiliary Boiler	Type 659	$Q_{aux}=\frac{\dot{m}c_{p}\left(T_{set}-T_{in}\right)+UA_{aux}\left(T_{aux}-T_{o}\right)}{\eta_{aux}}$
Stratified Thermal Tank	Type4a	$m_{i}C_{p}\frac{dT_{i}}{dT}={\dot{m}}_{i-1}C_{p}\left(T_{i-1}-T_{i}\right)-{\dot{m}}_{i+1}C_{p}\left(T_{i+1}-T_{i}\right)-UA_{i}\left(T_{i}-T_{amb}\right)$
Adsorption Chiller	Type 909	$Q_{chiller}={\dot{m}}_{chw}C_{p,chw}\left(T_{in}-T_{set}\right)$
Cooling Tower	Type51b	$NTU={c\left[\frac{{\dot{m}}_{a}}{{\dot{m}}_{w}}\right]}^{n+1}$

However, TRNSYS simulation software has some limitations that need to be consider. Building intricate systems can become challenging due to the complexity of connecting numerous components and ensuring proper data flow. Additionally, new users might find the interface unintuitive, requiring them to invest time in understanding the software despite available documentation. While extensive, the library of models within TRNSYS might not encompass every specific component you require for your simulation. Furthermore, the accuracy of simulations hinges on the chosen models and the quality of the data you input. It's crucial to be aware of these limitations to ensure you select the most appropriate models and parameters for your project.

**Fig. 1:**
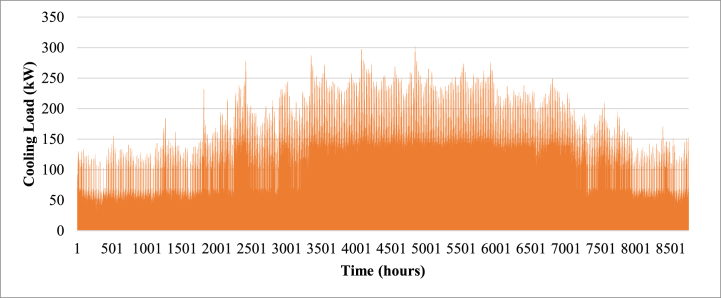
Annual cooling load.

**Fig. 2:**
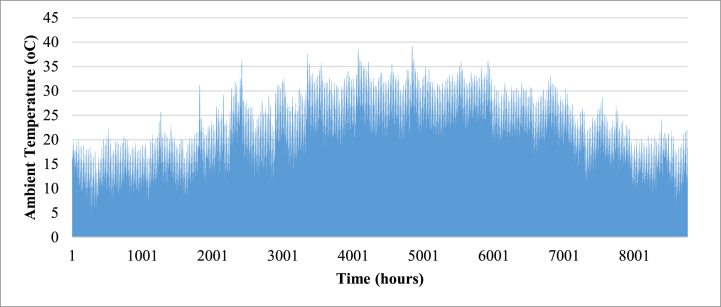
Annual ambient temperature.

The building model was developed using construction material specifications from Table 4 for external heat loads and internal heat loads from Table 5. The most important of these loads is the equipment internal heat gain by the Jacquard units which are presented in Table 6. It is necessary to maintain the zones containing the Jacquard units at 24 °C set point to avoid overheating and breakdown of equipment. The developed Type 65 building model is seamlessly incorporated into the TRNSYS simulation studio, and the city of Cairo is selected from the Meteonorm weather data library. The internal set temperature for each zone is established at 24 °C with a relative humidity of 50 %, aligning with the specifications outlined in the Egyptian Building Code (304). TRNbuild is equipped with a cooling/heating feature, allowing users to simulate the energy demand required to maintain the desired indoor temperature. To avoid hysteresis, a dead band of 2 °C is applied. Subsequently, the model undergoes a year-long simulation (8760 h) with 1-min intervals, revealing the cooling demand and ambient dry bulb temperature, as depicted in Fig. 1, Fig. 2 respectively.

**Table 4:** Construction materials specifications used in TRNBUILD.

Construction	Material	Area (m^2^)	Thickness (m)	U-value (W/m^2^K)
Internal walls	Wood	689.2	0.035	2.744
	Glass	499.5	0.035	4.878
Floor	Wood	464.7	0.1	1.378
Ceiling	Steel	464.7	0.03	5.859

**Table 5:** Internal heat gains used in TRNBUILD.

Internal heat gain	Description
Occupancy	Light Bench Work: 120 W per factory work according to ASHRAE Standard 90.1.
Infiltration	0.5 ACH according to Egyptian code for cooling and air conditioning (code 304.1)
Lighting	10 W/m2 according to the Egyptian lighting code (code 308)
Equipment	$Q=P_{rated}*SHF$ As outline in ASHRAE Fundamentals Handbook 2009, 50 % sensible heat factor should be assumed as a conservative estimation

**Table 6:** Jacquard units rated heat gain values.

Model	Zone	Prated (kW)	Operation hours (24 h/week)
VDW - CLX	1	7.106	8760
VDW - CLX	2	7.106	8760
Schonherr - alpha 300	3	7.106	8760
Schonherr - alpha 360	4	7.106	8760
VDW - CRX	5	7.106	8760
Schonherr - alpha 300	6	7.106	8760
Schonherr - alpha 300	7	7.106	8760
VDW - CRX	8	7.106	8760
VDW - CLX	9	7.106	8760
VDW - CRT	10	20.349	8760
VDW - SRX	11	7.106	8760
Schonherr - alpha 360	12	7.106	8760
VDW - CLX	13	7.106	8760
Schonherr - alpha 360	14	7.106	8760
VDW - SRX	15	7.106	8760
VDW - RC	16	7.106	8760
**Total**		**126.939**	8760
**Assuming SHF = 0.5**		**63.4695**	8760

Technical specifications for the solar collectors and adsorption chiller are based on manufacturer data from products available in the market as shown in Table 7, Table 8 respectively. The industrial building under investigation utilizes a Babcock-Wanson industrial boiler primarily for the shearing stage of the manufacturing process. However, the steam demand for these processes is relatively low, resulting in the boiler being oversized and generating excess steam. Specifically, other processes consume only 0.4 ton/h of steam, while the maximum steam flow rate of the boiler is 2.25 ton/h, as detailed in the technical specifications in Table 9 below.

**Table 7:** Hyrdosol VT5815 evacuated tube collector technical specifications [18].

Specification	Dimension
Gross Area	4.56 m^2^
Length	1.976 m
Number of Evacuated Tubes	30
Material	Oxygen Free copper (Tu1) Cu + Ag> 99.99 % (O2 <16 ppm)
Tube Dimensions	1800 mm length 14 mm outer diameter 0.75 mm thickness
Maximum Working Temperature	300 °C
Optical Efficiency Coefficient (a_o_)	0.42
1st Order Thermal Efficiency Coefficient (a_1_)	0.654 (W/m^2^.^o^C)
2nd Order Thermal Efficiency Coefficient (a_2_)	0.003 (W/m^2^.^o^C)

**Table 8:** Bryair E-100 adsorption chiller technical specifications [19].

Chiller model: E−100		
Nominal Capacity (TR)		104
Actual Capacity (TR)		85
Hot water	Inlet temperature (^o^C)	90.6
	Outlet temperature (^o^C)	85.2
	Water Flow rate (m^3^/h)	94.5
Chilled water	Inlet temperature (^o^C)	12
	Outlet temperature (^o^C)	7
	Water Flow rate (m^3^/h)	51.8
Cooling water	Inlet temperature (^o^C)	32
	Outlet temperature (^o^C)	38.4
	Water Flow rate (m^3^/h)	120

**Table 9:** Babcock-Wanson 200 GS boiler technical specifications.

Brand	Babcock-Wanson
Model	200 GS
Operating Fuel	Natural Gas
Maximum pressure (bar)	12
Maximum flowrate (kg/h)	2250
Efficiency	0.7

**Fig. 3:**
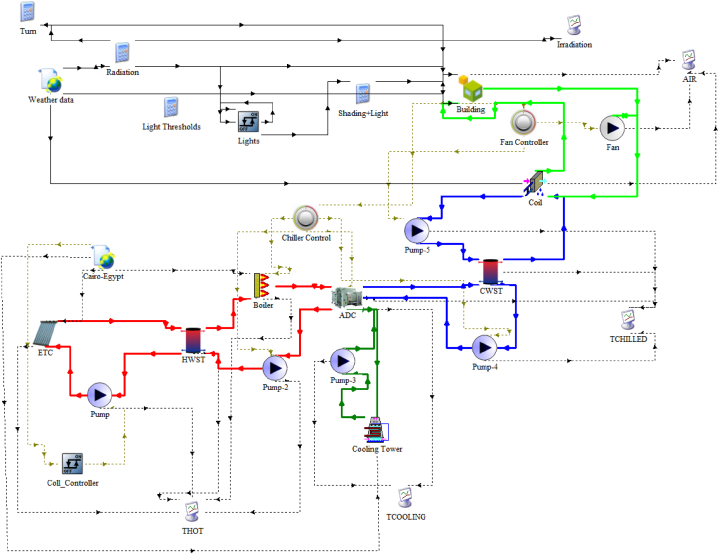
Final TRNSYS solar assisted adsorption chiller system model.

Fig. 3 shows the final system modelled using the TRNSYS simulation software to cater to the cooling energy requirements of an industrial building under the weather conditions in Cairo, Egypt. The system is intricately divided into three subsystems, each meticulously managing specific cycling fluids. The first subsystem, the hot water subsystem, encompasses the pathway from solar collectors to the storage tank, incorporating the hot water cycle from the storage tank to the adsorption chiller through the auxiliary heater. The second subsystem, the cooling water subsystem, spans from the adsorption chiller to the cooling tower. Finally, the third subsystem, the load subsystem, connects from the adsorption chiller to the fan coil units and building load. Each subsystem's operation is meticulously governed by controllers.

As shown in Fig. 3, the hot water subsystem consists of two pumps. The solar pump is constant speed providing constant flowrate through the solar collector field. A variable speed pump is utilized between the hot water storage tank, auxiliary boiler, and adsorption chiller to supply variable flow rates based on rate based on the heat energy required by the chiller to produce the required cooling power at any moment in the simulation. This variable flow is then circulated through the hot water storage tank to extract the heat energy provided by the solar collectors, and then is compensated by the boiler to reach outlet temperature of 90.6 °C.

## 4 Model validation

The validation of our model involves comparing simulation results, specifically temperatures of chiller inlet/outlet, hot water, cold water, and chilled water, with data from previous studies that have examined similar systems. It should be noted that the components in these systems may differ in size from those in our proposed model, including solar collector area, storage tank capacity, and chiller capacity. To ensure rigorous validation, adjustments are made to our model's components to match those of the referenced systems. Importantly, the overall configuration of components in our model remains consistent with the original proposal as depicted in Fig. 3. The comparison methodology includes assessing either a single-point comparison, focusing on average or maximum parameter values or an hourly comparison of these parameters for a particular day. The selection of approach depends on the data availability from the published works used in the validation process. This rigorous validation procedure guarantees the reliability of our proposed model, providing a thorough and detailed evaluation against a wide range of comparable systems.

Table 10 presents a comparison of temperatures within our simulation model, including those of the solar collector outlet, chiller hot water inlet, chilled water outlet, and fan coil inlet. When compared with both experimental and simulated results from a referenced study [20], our model demonstrates close alignment with the published experimental data, showing a maximum Mean Bias Error (MBE) of 9 %. These findings indicate that our model performs effectively in this comparison and receives validation from the referenced study, which utilized a system with similar components and sizes. As a result, we can rely on our proposed model to accurately predict the performance of an actual solar thermal cooling system, even when the component sizes vary slightly from those specified in our model.

**Table 10:** Experimental/simulation results comparison [20].

System outputs	Between 10:00 and 16:00			
	Average Temperature (^o^C)			
	[20] Simulation	[20] Paper Experimental	Proposed Simulation	MBE
Collector outlet temp	84.7	81.7	83	2 %
Chiller Hot water inlet temp	82.2	78.0	81	4 %
Chilled water outlet temp	10.9	9.8	10.7	9 %
Fan coil inlet temp	11.0	10.5	9.9	6 %

**Fig. 4:**
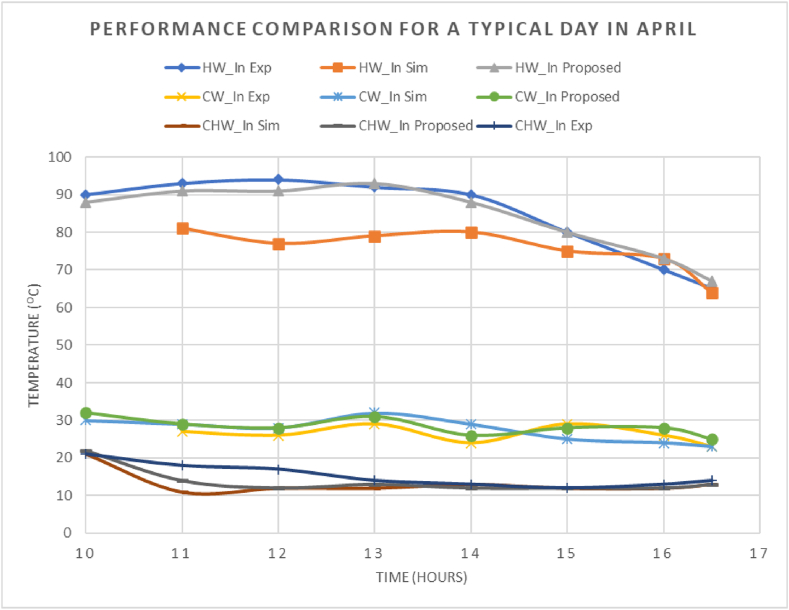
Hourly experimental/simulation data comparison [21].

Fig. 4 illustrates that the hourly hot water inlet temperatures into the chiller in our proposed model closely match the experimental data [21], with a Mean Bias Error (MBE) of 2 % for that specific day. Similarly, our model accurately predicts the hourly temperatures of cold water entering the chiller, closely aligning with both experimental and simulation results from the referenced study, achieving an MBE of less than 7 %. Regarding chilled water exiting the chiller, both the simulation model from the referenced study [21] and our proposed model exhibits comparable behavior throughout the day. However, it is noted that experimental results [21] take longer to reach the desired chilled water temperature, resulting in higher deviations from simulation during the initial 3 h until stabilization around 12 °C. Considering these initial hours as outliers, our proposed model achieves an MBE of 6 %, indicating accurate prediction of hourly temperatures.

**Fig. 5:**
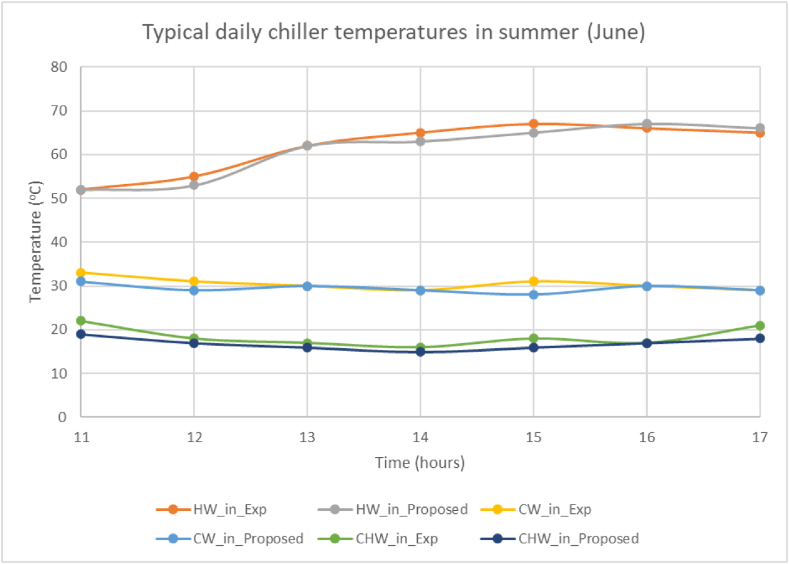
Hourly experimental/simulation data comparison [22].

Fig. 5 illustrates the comparison between the chiller's inlet and outlet temperatures, showcasing the alignment between the experimental measurements [22] and the predictions from the proposed model simulation. Notably, both systems exhibit analogous trends in the inlet hot water temperatures, with a Mean Bias Error (MBE) of 2 %. Similarly, the inlet cold temperatures from the experimental measurements [22] and the proposed model display comparable trends, with an MBE of less than 4 %, while the inlet chilled temperatures show a slightly higher MBE of 8 %, however still within the acceptable range of ±10 % for hourly data.

## 5 Results and discussion

A comprehensive analysis of the solar adsorption cooling system's performance under the climatic conditions of 10th of Ramadan, Cairo, Egypt, was conducted through a year-long simulation with hourly intervals, totalling 8760 h, using the TRNSYS simulation tool. This study, focused on identifying the optimal system configuration by examining crucial parameters like collector area, hot water storage tank (HWST) volume, and cold-water storage tank (CWST) volume. The energetic metrics, namely solar fraction was assessed based on TRNSYS simulation results. The Evacuated Tube Collectors' (ETCs) collector array varied from 50 m^2^ to 200 m^2^, and the capacities of hot water and cold-water storage tanks were adjusted within the range of 1 m^3^–10 m^3^. It was crucial to maintain the hot water entering the adsorption chiller at 90.6 °C to ensure optimal operation of the chiller, aligning with the manufacturer's recommendations.

A. Collector Area Optimization

**Fig. 6:**
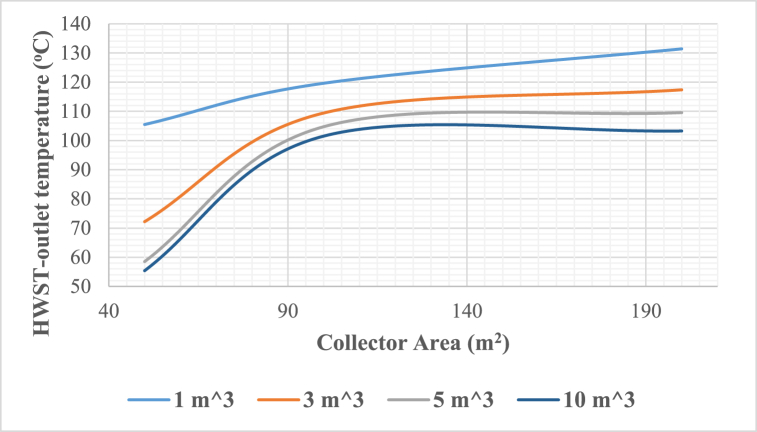
Hot water storage tank outlet temperature vs collector area at 3 m^3^ cold water storage tank volume.

The influence of varying the collector area on the outlet temperature of the hot water storage tank is interpreted in Fig. 6. The findings suggest that an area surpassing 100 m^2^ does not contribute to an increase in tank outlet temperatures, except for a 1 m^3^ storage tank volume, which exhibits temperatures surpassing 100 °C at atmospheric pressure, leading to undesired pressurization. Systems manifesting such characteristics are undesirable, posing potential hazards for domestic use and potential harm to both the system and its components. The optimal collector field area to supply the adsorption chiller with the required hot water temperature for operation is between 80 and 90 m^2^, aligning with the hot water inlet set temperature of 90.6 °C.

**Fig. 7:**
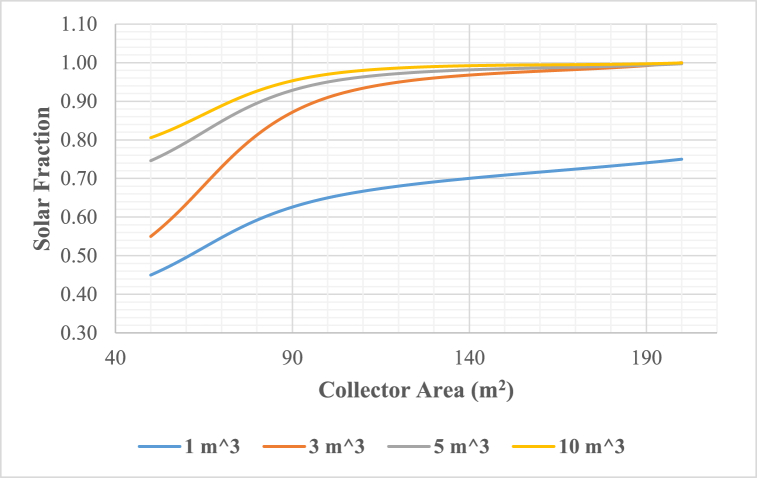
Solar fraction vs collector area at 3 m^3^ cold water storage tank volume.

The maximum solar fraction of the proposed adsorption cooling systems exhibits a noticeable increase with the expansion of the solar collector field area, reaching a maximum value of 1.0. However, beyond this point, further increments yield no additional benefits. As illustrated in Fig. 7, the impact of solar collector area on the solar fraction is evident; increasing the collector area from 50 to 100 m^2^ results in a substantial change in maximum solar fraction, increasing from 0.58 at 1 m^3^ HWST volume to 1.0 at 10 m^3^ HWST volume respectively. Yet, extending the area from 100 m^2^ to 200 m^2^ shows no effect on maximum solar fraction, as it has already reached the maximum threshold at a 100 m^2^ collector area. Any additional collector area has no impact on both solar fraction and boiler energy consumption, potentially leading to thermal energy overproduction, significantly affecting the initial investment costs of the entire cooling system.

B. Hot Water Storage Tank Optimization

**Fig. 8:**
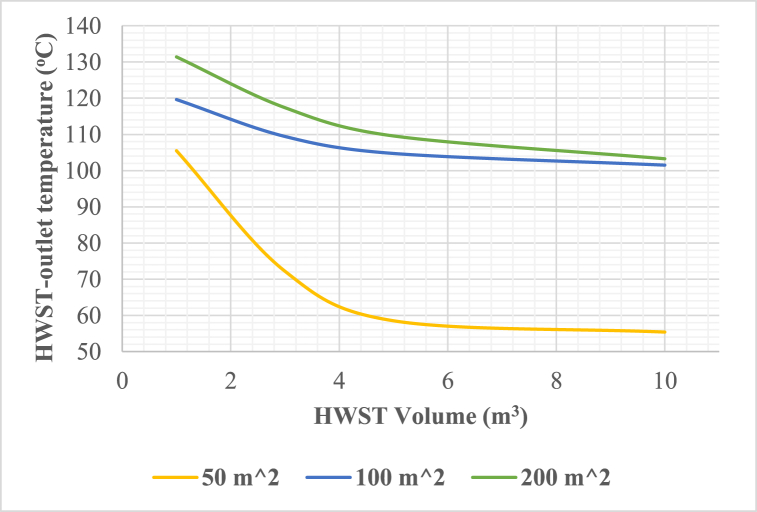
Hot water storage tank outlet temperature vs hot water storage tank volume at 3 m^3^ cold water storage tank volume.

The influence of varying the hot water storage tank (HWST) volume on the HWST outlet temperature is depicted in Fig. 8. The findings suggest that an HWST volume surpassing 4 m^3^ has no impact on the tank outlet temperatures. On the contrary, smaller storage tanks exhibit a deficiency in storing sufficient heat, resulting in an increased reliance on the auxiliary heater. It is evident that enlarging the hot water storage tank beyond 4 m^3^ does not provide any notable advantage. Consequently, increasing the HWST volume beyond this threshold would only escalate the initial investment costs of the system.

**Fig. 9:**
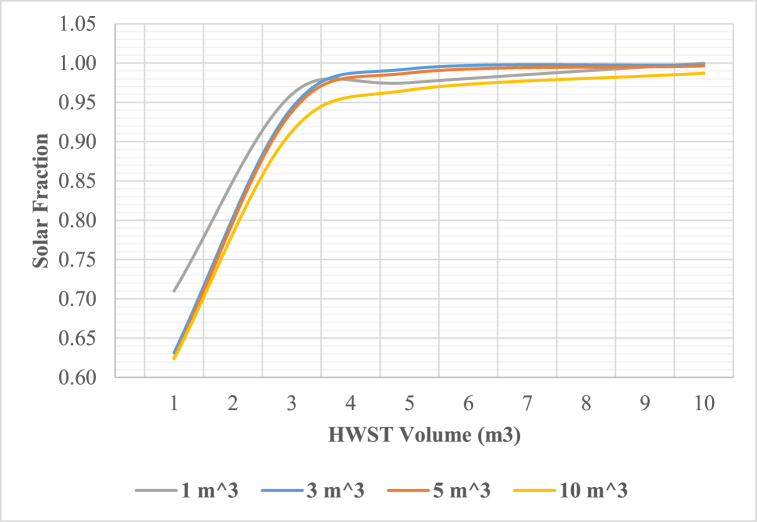
Solar fraction vs hot water storage tank volume at 100 m^2^ collector area.

Fig. 9 visually represent the impact of hot water tank volume on the solar fraction. Remarkably, the solar fraction demonstrates an increase from 0.71 to 0.99 as the storage tank volume varies from 1 to 4 m^3^ at collector area of 100 m^2^. However, beyond the 4 m^3^ threshold, any further expansion of the storage tank volume exhibits minimal influence on both the solar fraction, rendering such enlargement not advisable.

C. Cold Water Storage Tank Optimization

Results show that adjusting the CWST volume has negligible to no impact on the outlet temperature of the HWST outlet temperature. Nevertheless, elevated temperatures are observed at smaller HWST volumes and larger collector areas, with a peak outlet temperature of 183 °C at 1 m^3^ HWST volume and 200 m^2^ collector area. CWST volume exerts minimal influence on HWST outlet temperatures as they remain relatively constant across the different CWST volumes. Furthermore, the impact of varying CWST volume on solar fraction at 50 m^2^ findings reveal a decrease in solar fraction, ranging from 0.58 to 0.92 at 1 m^3^ CWST volume, to a range of 0.47–0.76 at 3 m^3^. However, beyond this value, solar fraction is minimally affected by changes in storage tank capacity and remains relatively constant. For collector areas of 100 and 200 m^2^, the incremental increase in solar tank capacity has no discernible effect on the solar fraction. The results demonstrate a maximum solar fraction of 1.0 for both 100 and 200 m^2^ with HWST volumes above 1 m^3^ across all CWST volume values. Additionally, these findings suggest that solar fraction sensitivity to CWST volumes is more pronounced at smaller collector areas, attributed to the inability of smaller collector areas to drive the adsorption chiller for producing the required cooling energy demanded by the CWST.

The results of the parametric analysis demonstrate the importance of determining the optimal values for collector area and storage tank volume based on factors such as maximum solar fraction and minimizing boiler energy requirements. Given that the size of the solar collectors has significant financial implications, the ideal collector area was determined with a focus on maximizing the solar fraction. However, it's crucial to also consider the outlet temperature of the hot water storage tank (HWST) to ensure that hot water is delivered to the adsorption chiller at the required temperatures. Regarding the storage tank volume, increasing the volume leads to a higher solar fraction initially. However, beyond a certain point, the increase in solar fraction becomes negligible with further increases in volume. Therefore, selecting a larger volume beyond this specific threshold is unnecessary. This threshold volume is considered as the optimal value for the storage tank. The optimal sizes of the components determined through the parametric analysis are presented in Table 11.

D. Thermal power of the solar thermal system

**Table 11:** Design parameters optimal configuration values.

Parameter	Optimal value
Collector Field Area	90 m^2^
Hot water storage tank volume	4 m^3^
Cold water storage tank volume	3 m^3^

**Fig. 10:**
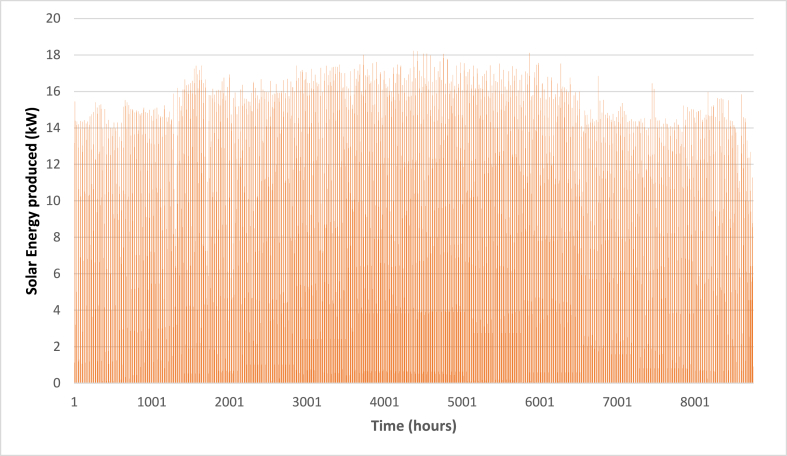
Hourly variation of solar thermal power (AC = 50 m^2^).

**Fig. 11:**
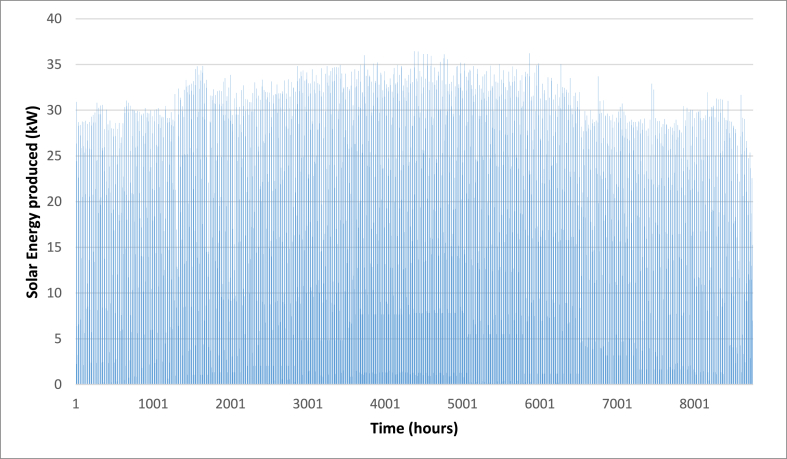
Hourly variation of solar thermal power (A_C_ = 100 m^2^).

**Fig. 12:**
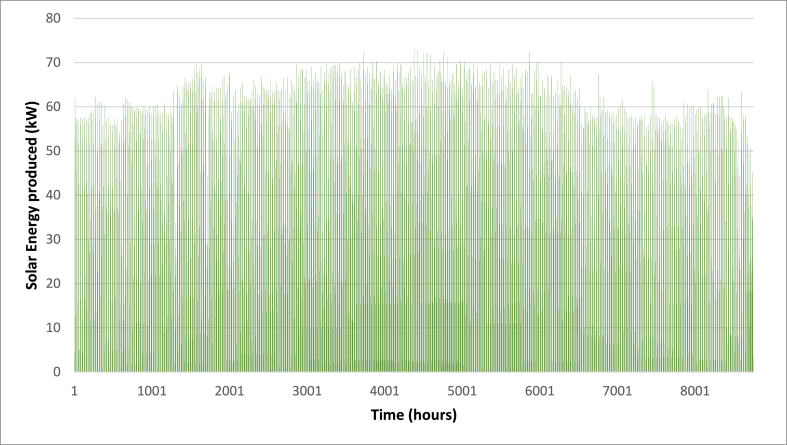
Hourly variation of solar thermal power (A_C_ = 200 m^2^).

Below are Fig. 10, Fig. 11, Fig. 12 depicting the results of the hourly variation in thermal power output from the solar thermal system. These figures illustrate how the thermal power varies across different collector area configurations: specifically, 50 m^2^, 100 m^2^, and 200 m^2^. At these respective areas, the solar thermal system achieves peak thermal power outputs of 18.2 kW, 36.5 kW, and 72.9 kW. Fig. 9 showcases the hourly thermal power output when the solar collector area is set at 50 m^2^. It demonstrates that the system reaches its maximum thermal power output of 18.2 kW during peak periods. Fig. 10 expands upon this analysis by presenting the thermal power variation with a larger collector area of 100 m^2^. Here, the system achieves a higher peak thermal power output of 36.5 kW, illustrating the increased efficiency and capacity of the system with a larger collector area. Fig. 11 further extends this analysis to a solar collector area of 200 m^2^, showing the highest peak thermal power output of 72.9 kW. This figure highlights the substantial enhancement in thermal power generation capability when the collector area is doubled from the 100 m^2^ configuration.

E. Thermal power of the gas boiler

**Fig. 13:**
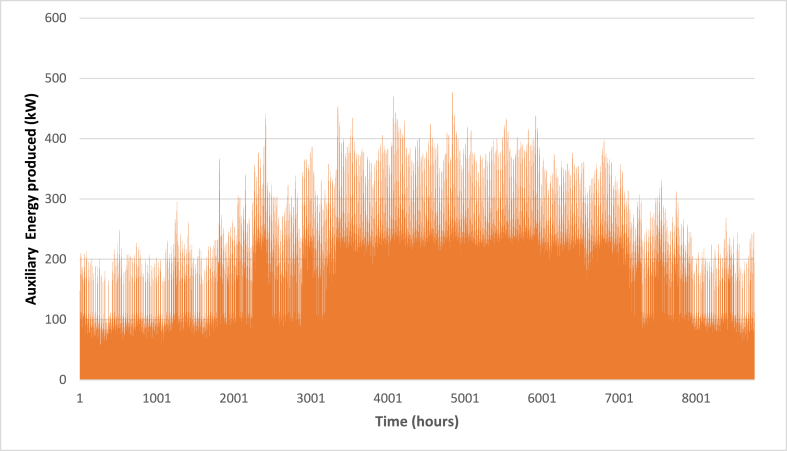
Hourly variation of auxiliary power (A_C_ = 50 m^2^).

**Fig. 14:**
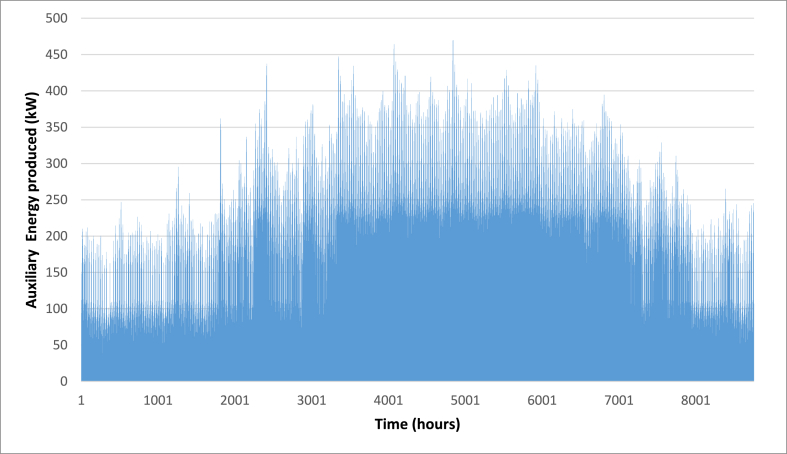
Hourly variation of auxiliary power (A_C_ = 100 m^2^).

**Fig. 15:**
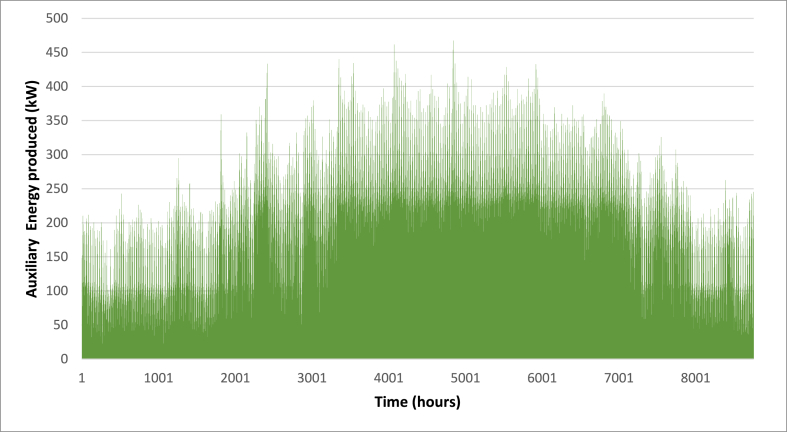
Hourly variation of auxiliary power (A_C_ = 200 m^2^).

Fig. 13, Fig. 14, Fig. 15 depict the hourly variation in auxiliary power output from the gas boiler. These figures demonstrate how auxiliary power fluctuates across different collector area sizes: specifically, 50 m^2^, 100 m^2^, and 200 m^2^. Fig. 9 illustrates the hourly auxiliary power output when the solar collector area is 50 m^2^, showing that the system reaches its maximum auxiliary power output of 477 kW during peak periods. Fig. 10 builds on this analysis by displaying the auxiliary power variation with a larger collector area of 100 m^2^. In this configuration, the system achieves a higher peak auxiliary power output of 470 kW, indicating improved efficiency and capacity compared to the 50 m^2^ setup. Fig. 11 extends this examination to a solar collector area of 200 m^2^, revealing the lowest peak auxiliary power output of 467 kW. These figures provide a clear depiction of how the gas boiler's hourly auxiliary power output varies across different collector area sizes, highlighting the system's scalability and its enhanced performance with larger collector areas.

F. Economic Assessment

Following the completion of parametric analysis, a comprehensive economic assessment is conducted. This aims to ascertain the financial feasibility of implementing the proposed solar adsorption cooling system in an industrial building in Egypt. Table 12 contains crucial information such as the latest adjusted electricity tariff, unit price of natural gas, discount rates, system lifetime, and specific costs of the system components, which will be utilized in the economic analysis.

**Table 12:** Input data values used in economic assessment.

Component	Unit	Value
HYDROSOL ETC cost [23]	$/m2	288.00
Storage Tank cost [24]	$/m3	300.00
100 TR BryAir Adsorption Chiller cost [25]	$	170,000.00
Cooling Tower price [26]	$/kW	16.00
Electricity Tariff [27]	$/kWh	0.04
Natural gas price [28]	$/mmBTU	4.75
Discount rate	%	19.75 %
System Lifetime	Years	25
Performance Degradation	% per year	1

The proposed solar adsorption system's cost was categorized into six components: collector cost, adsorption chiller cost, auxiliary heater cost, storage tanks cost, and operating cost. In the optimal design scenario, the solar collector's cost constituted 10 % of the total, while the adsorption chiller's cost made up 67 % of the total cost. The auxiliary heater's cost was excluded since the industrial building already has a boiler that will be fully utilized in implementing this system. The initial investment in equipment accounted for 86 % of the total cost, with operation costs representing only 14 %.

### 5.1 Payback period (PBP)

By implementing the proposed solar adsorption cooling system in an industrial building in Cairo, Egypt, annual savings of approximately 27,000 USD are achieved. This amount represents the annual electricity bill for operating conventional air conditioning systems, which is the current practice in Egypt. Consequently, the payback period for implementing the proposed solar absorption cooling system in an industrial building in Egypt is estimated to be around 7.4 years.

### 5.2 Net present value (NPV)

The net present value (NPV) and internal rate of return (IRR) calculations were conducted using three discount rates: 5 %, 10 %, and 20 %, considering a project lifespan of 20 years. The IRR serves as a metric for assessing investment profitability, where higher IRR values indicate more favorable investment opportunities. An investment is considered profitable when the IRR surpasses the risk threshold. Results indicate a positive NPV of 38,890 USD at a 10 % discount rate, and negative NPVs of −25,980 and −65,363 USD at 15 % and 20 % discount rates, respectively. The IRR is calculated to be 12.85 %, suggesting that the implementation of the proposed solar-assisted cooling system is financially viable when the discount rate is lower than the IRR. However, there has been an increase in Egypt's discount rates, rising from 9.75 % in March 2022 to 19.75 % in December 2023, with an anticipated upward trend in the future. Therefore, it is crucial to assess the economic feasibility of the proposed system under the current discount rate of 19.75 % using the NPV method. The results indicate a negative NPV, indicating that the proposed system is not financially viable at the current discount rate in Egypt. This holds true if the discount rate remains at 19.75 % or increases over the system's lifespan.

### 5.3 Return on investment (ROI)

The ROI formula typically entails dividing the net profit generated by the project by the initial investment cost and expressing it as a percentage. A positive ROI indicates that the project is generating returns, while a negative ROI suggests that the investment is not yielding sufficient financial gains. Results show the proposed system demonstrates an ROI of 34.5 % over a 10-year period.

### 5.4 Environmental impact

This section concentrates on assessing the environmental impact of the proposed solar adsorption system by quantifying the total decrease in CO_2_ emissions resulting from grid electricity usage and natural gas burning throughout the system's lifespan. The optimized parameters derived from the parametric analysis were utilized to compute the lifetime CO_2_ emissions. Egypt's Global Emission Factor (GEF) stands at 0.533 tons of CO_2_ per megawatt-hour (tCO2/MWh), with an annual consumption of 7967.2 GW-hours (GWh) of electricity specifically for air conditioning in buildings. The CO_2_ emission factor, derived from natural gas, is 52.91 kg of CO_2_ per million British thermal units (kgCO_2_/mmBTU). The findings indicate that the proposed system has the potential to reduce around 7,200 tons of CO_2_ emissions.

## 6 Conclusions

A solar-assisted adsorption chiller was investigated for application in an industrial building located in Cairo, Egypt. Utilizing detailed modeling and simulation, the study assessed both the system's thermal performance and economic viability. The analysis identified an optimal collector area of 90 m^2^ and a hot water storage tank size of 4 m³. The economic evaluation projected a favorable payback period of 7.6 years, an internal rate of return (IRR) of 14.3 %, and a return on investment (ROI) of 34.5 % over a 10-year period. Additionally, the system has the potential for significant CO2 emission reductions. The study acknowledged practical challenges such as space constraints and integration with existing systems. To address these challenges, the importance of advanced control algorithms and well-defined maintenance strategies was emphasized. Recommendations for future research include refining the economic analysis, investigating system adaptability to varying electricity pricing structures, and exploring the potential of hybrid solar systems for further performance improvements.

## 7 Practical challenges

Implementing solar-powered cooling systems in industrial buildings presents various practical challenges and operational considerations that must be addressed for a successful implementation. Limited space for solar panel installation is a common issue, necessitating careful planning regarding the space requirements for solar collectors and supporting equipment like storage tanks and heat exchangers. Integrating these systems with existing industrial processes or HVAC setups can be intricate, involving considerations such as compatibility, control methods, and system optimization, which demand thorough engineering expertise.

Industrial sites experience fluctuating cooling demands and load profiles, requiring the solar-powered cooling system to efficiently adapt to these changes. This necessitates the use of advanced control algorithms and predictive modeling to enhance energy utilization and overall system performance. Reliability is crucial in industrial settings, prompting the need for redundancy measures, backup power alternatives, and robust maintenance strategies to address potential failures or disruptions in solar energy supply.

Adhering to local regulations, building codes, and environmental standards is essential during the installation phase, involving tasks such as obtaining permits, navigating zoning requirements, and ensuring compliance with safety regulations. Furthermore, ongoing maintenance, performance monitoring, and optimization play a vital role in sustaining the effectiveness and efficiency of solar-powered cooling systems over time. Proactive measures like implementing maintenance schedules, tracking energy usage, and analyzing performance data are instrumental in detecting issues early and maximizing system dependability.

Successfully managing these practical challenges and operational considerations requires collaborative efforts among engineers, architects, energy specialists, and facility managers to design, implement, and oversee solar-powered cooling systems effectively within industrial buildings.

## 8 Recommended future works

Several recommendations can be proposed for future investigations into the feasibility of implementing solar adsorption chiller systems, building upon the insights gained from this study. Refining the economic dimensions of the system through sensitivity analyses on various economic factors such as interest rates or maintenance costs. This approach would yield a more comprehensive understanding of the system's economic feasibility. Given the relatively low electricity prices in Egypt compared to other global regions, exploring how the system's design and functionality might adapt in diverse areas with differing electricity price frameworks would be intriguing. This could entail conducting case studies across various countries or regions to assess how electricity prices influence system optimization and to identify the most cost-effective solutions. Furthermore, investigating the potential of hybrid solar systems that integrate both solar photovoltaic and solar thermal collectors presents an avenue for further research. By optimizing the design parameters of such hybrid systems using a similar analytical framework, researchers can ascertain the most effective configuration and operational strategy to maximize solar energy utilization and enhance overall system performance.

## CRediT authorship contribution statement

**Mohammed A. Ebaid:** Writing – original draft, Software, Methodology, Investigation, Formal analysis. **Tamer A. Mohamed:** Writing – review & editing, Supervision, Resources. **Hesham Safwat:** Writing – review & editing, Supervision, Conceptualization.

## Declaration of competing interest

The authors declare that they have no known competing financial interests or personal relationships that could have appeared to influence the work reported in this paper.

## Figures and Tables

**Table:** 

A_c_	Surface area of the collector
A	Tank surface area
C_p_	Fluid specific heat capacity
C_o_	Total Initial Investment
C_F_	Annual Cashflow
C_i_	Project Annual Cashflow at year i
C_net_	Net return on investment
CE_E_	CO_2_ emissions from electricity
CE_NG_	CO_2_ emissions from natural gas
C_E_	electricity consumption
C_NG_	natural gas consumption
F_E_	electricity grid emission factor
F_NG_	natural gas emission factor
$\dot{m}$	Mass flow rate
n	Project Lifetime
$I_{T}$	Total Irradiation received by the collector
$Q_{aux}$	Auxiliary Energy Gain
$Q_{solar}$	Useful Energy Gain
r	Discount rate
T_amb_	Ambient Temperature
T_i_	Inlet Fluid Temperature
T_o_	Outlet Fluid Temperature
T_S_	Sun Temperature
T_set_	Set temperature
ΔT	Temperature difference between the outlet and the inlet
t	Time
U_L_	Overall heat loss coefficient of the solar collector
**Greek Symbols**	
τ	Transmissivity
α	Absorptivity
ε	Emissivity
ρ_w_	Density of Water
μ_w_	Dynamic Viscosity of Water
η	Energy Efficiency
β	Slope of the collector (Inclination Angle)
$\beta^{\prime }$	Volumetric coefficient of expansion
ϕ	Volume Fraction
g	universal gravitational constant
θ	Latitude
v	Kinematic Viscosity.
$\alpha^{\prime }$	Thermal Diffusivity
